# A case report of persistent drug-sensitive pulmonary tuberculosis after treatment completion

**DOI:** 10.1186/s12879-022-07836-y

**Published:** 2022-11-19

**Authors:** Sergo A. Vashakidze, Abivarma Chandrakumaran, Merab Japaridze, Giorgi Gogishvili, Jeffrey M. Collins, Manana Rekhviashvili, Russell R. Kempker

**Affiliations:** 1 Thoracic Surgery Department, National Center for Tuberculosis and Lung Diseases, 50 Maruashvili, 0101 Tbilisi, Georgia; 2 The University of Georgia, Tbilisi, Georgia; 3 Tbilisi State Medical University, Tbilisi, Georgia; 4 Division of Infectious Diseases, Department of Medicine, Emory University School of Medicine, Atlanta, GA USA

**Keywords:** Cavitary, Tuberculosis, Thoracic surgery, Persistent, Case report

## Abstract

**Background:**

*Mycobacterium tuberculosis* (Mtb) has been found to persist within cavities in patients who have completed their anti-tuberculosis therapy. The clinical implications of Mtb persistence after therapy include recurrence of disease and destructive changes within the lungs. Data on residual changes in patients who completed anti-tuberculosis therapy are scarce. This case highlights the radiological and pathological changes that persist after anti-tuberculosis therapy completion and the importance of achieving sterilization of cavities in order to prevent these changes.

**Case presentation:**

This is a case report of a 33 year old female with drug-sensitive pulmonary tuberculosis who despite successfully completing standard 6-month treatment had persistent changes in her lungs on radiological imaging. The patient underwent multiple adjunctive surgeries to resect cavitary lesions, which were culture positive for Mtb. After surgical treatment, the patient’s chest radiographies improved, symptoms subsided, and she was given a definition of cure.

**Conclusions:**

Medical therapy alone, in the presence of severe cavitary lung lesions may not be able to achieve sterilizing cure in all cases. Cavities can not only cause reactivation but also drive inflammatory changes and subsequent lung damage leading to airflow obstruction, bronchiectasis, and fibrosis. Surgical removal of these foci of bacilli can be an effective adjunctive treatment necessary for a sterilizing cure and improved long term lung health.

## Background

Mycobacterium tuberculosis treatment has been evolving over the years, especially with the introduction of newer drugs and shorter regimens [1, 2]. Apart from the cavitary nature of tuberculous disease, patients who have been treated with current regimens often are given the designation of cure without achieving proper sterilization. Patients who complete the tuberculous regimen are given the definition of cure after they achieve sputum negativity but many of these patients harbor bacilli within cavities that continue to exert their effects on the respiratory system [3]. The residual changes that occur in patients who have completed medical therapy have been poorly attended to in the literature. Patients that underwent surgical and medical sterilization have been reported to have better pulmonary health in the long term, especially after the removal of cavities [4].

Here, we report a patient who underwent a complete regimen of medical therapy for pulmonary tuberculosis and later had to have surgical resection of her cavities, which grew tuberculous bacilli even after achieving sputum negativity.

## Case presentation

**Fig. 1:**
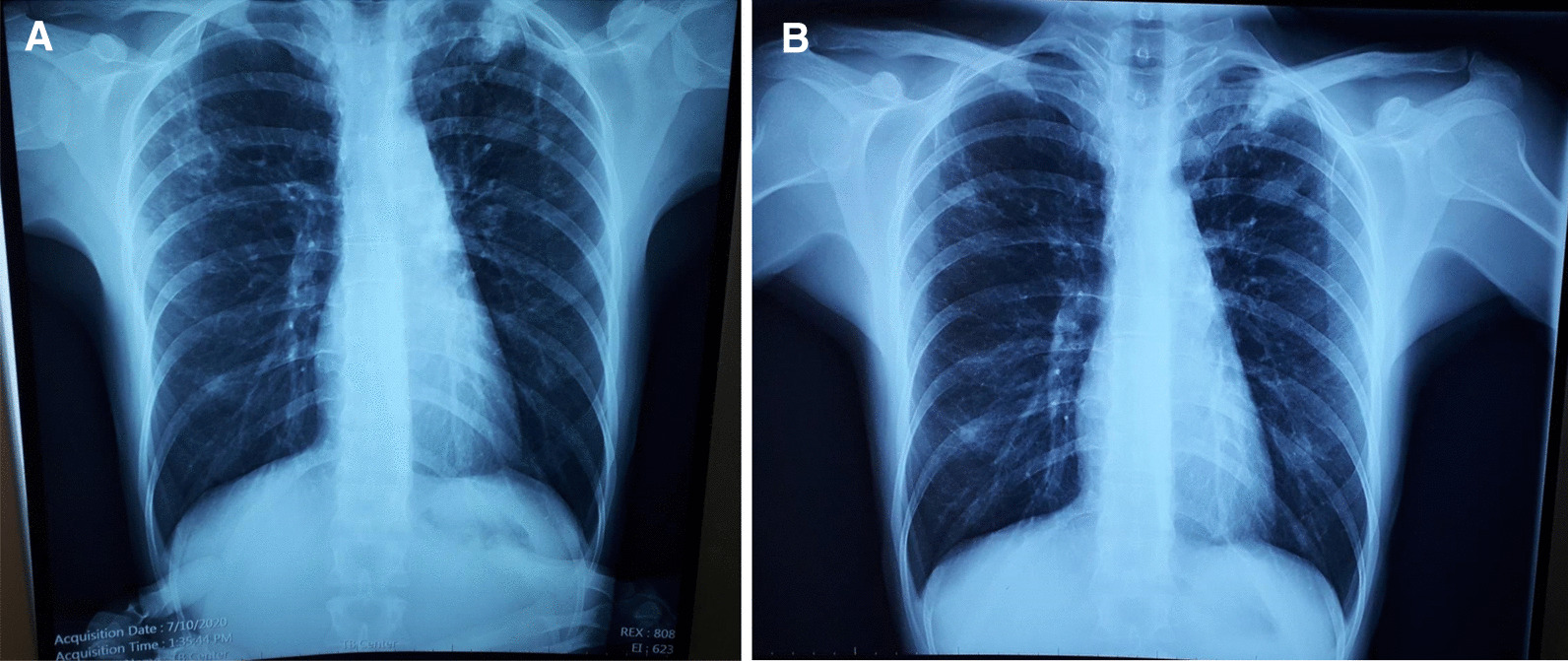
**A** (left): Baseline chest X-ray showing a cavity in the right lung and multiple foci in the upper lobes of both lungs. **B** (right): End of initial treatment chest X-ray, showing fibrosis, local honeycombing and dense focal shadows in both lungs

**Fig. 2:**
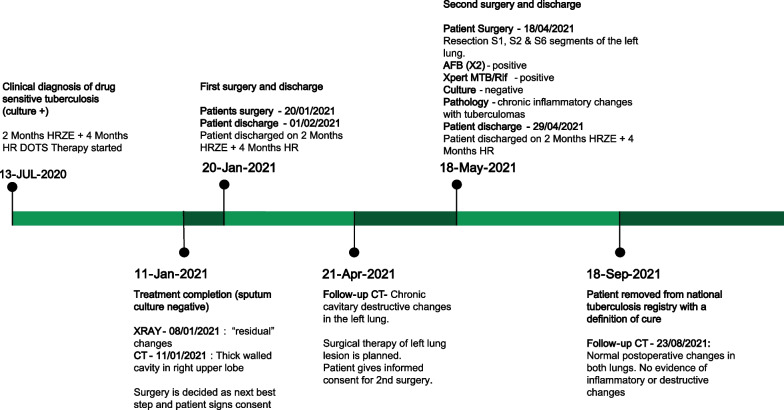
Patient treatment timeline (*HRZE* isoniazid, rifampin, pyrazinamide, ethambutol; *HR* isoniazid & rifampin; *DOTS* directly observed therapy, short-course; *CT* computed tomography; *AFB* acid fast bacilli)

A 33-year-old female from the country of Georgia presented to a tuberculosis dispensary on July 10, 2020, with a temperature of 38° C and symptoms of malaise, productive cough, and night sweats. The patient had no known medical problems. She reported smoking ~ 10 cigarettes daily and denied alcohol or illicit drug use. She had 3 children and her husband was a prisoner being treated for pulmonary tuberculosis. Upon physical examination there were decreased breath sounds in the upper lobes of the lungs with dullness to percussion. The patient had a body mass index (BMI) of 16.3 kg/m^2^. A complete blood count revealed a moderate leukocytosis of 10.2 × 10^9^/L and an erythrocyte sedimentation rate (ESR) of 42 mm/h. Biochemical blood parameters were normal. Sputum testing found a negative acid-fast bacilli (AFB) microscopy, positive Xpert MTB/RIF test (no RIF resistance), and positive culture for *Mycobacterium tuberculosis* (Mtb). Additionally, drug susceptibility testing (DST) revealed sensitivity to rifampin, isoniazid, and ethambutol. Chest radiography revealed multiple small foci in the upper lobes of both lungs and a cavity in the right lung (Fig. 1A). The patient was initiated on daily outpatient treatment with three pills of a fixed dosed combination pill containing isoniazid 75 mg, rifampin 150 mg, ethambutol 275 mg and pyrazinamide 400 mg. Treatment was given through directly observed therapy (DOT). She converted her sputum cultures to negative at 2 months and continued rifampin and isoniazid to finish 6 months of treatment. An end of treatment chest x-ray revealed fibrosis and honeycombing in the right upper lung, and fibrosis and dense focal shadows in the 1st and 2nd intercostal spaces of the left lung (Fig. 1B). The complete treatment timeline is summarized in Fig. 2.

**Fig. 3:**
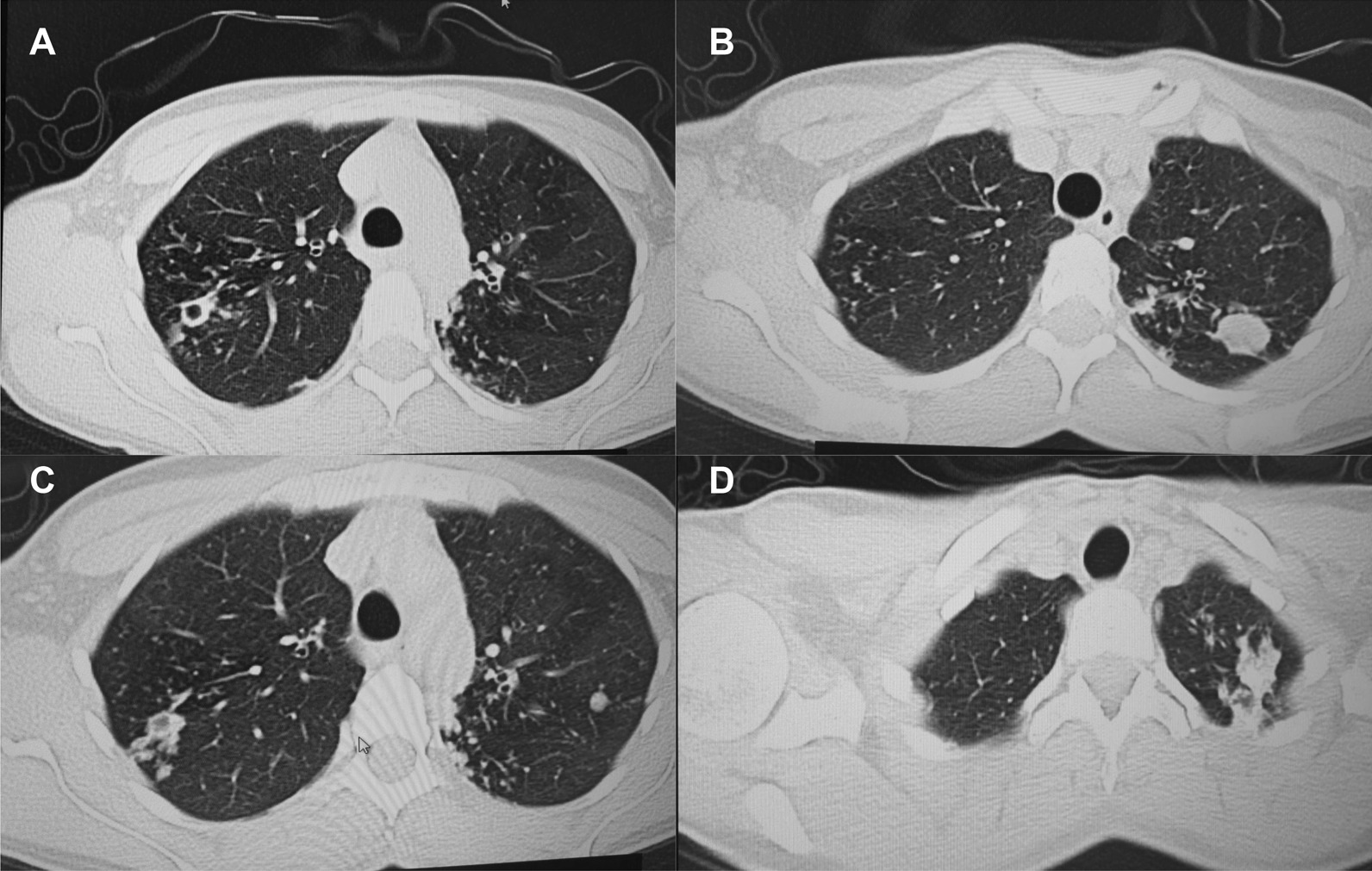
CT scan (January 11, 2021) showing, **A** a cavity in the upper lobe of the right lung with heterogeneous thick walls. **B** S1 and S2 segments of the left lung shows a 23 × 18 mm oval shaped calcified inclusions; **C**, **D** areas with calcified, compacted nodules 13 × 20 mm in size with additional traction bronchiectasis

**Fig. 4:**
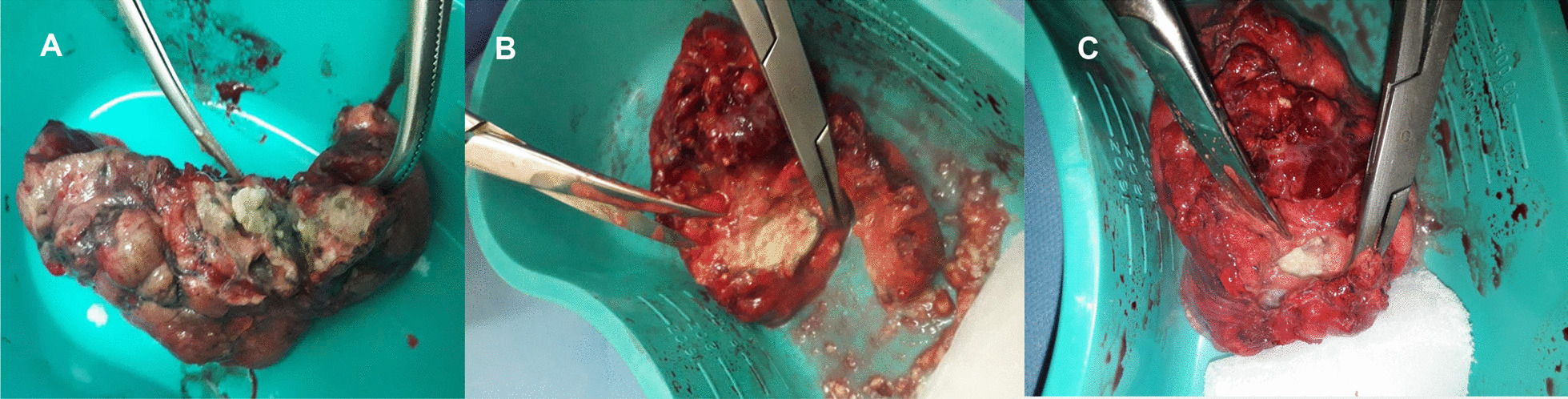
**A** Gross pathological image of a resected cavity with caseous material from first surgery (S1 & S2 segment of right lung). **B** The gross pathology from the second surgery showed the presence of a blocked cavity measuring up to 2 cm in diameter filled with caseous material in the S1, S2 and **C** Tuberculoma in S6 segment

A follow up chest computed tomography (CT) scan demonstrated a cavity in the right upper lobe measuring 12 × 10 mm in size with a thick and heterogeneous wall and nodules and bronchiectasis in the left lung (Fig. 3A–D). Based on CT findings and in accordance with National tuberculosis guidelines, the patient was offered surgical resection of the affected portion of the lung. It should be noted that the patient reported no symptoms, complaints, or functional disability before the surgery. Preoperative workup including pulmonary function testing, an echocardiogram, bronchoscopy, and blood chemistries were normal. The patient consented to surgery and underwent a surgical resection of the S1 and S2 segments of the right lung 2 weeks later. Intraoperatively, moderate adhesions were visualized in the S1 and S2 area with a palpable dense formation ~ 3.0 cm in diameter, in addition to a dense nodule. Gross pathology of the resected lesion showed a thick-walled fibrous cavity filled with caseous necrosis (Fig. 4A) corresponding to the right preoperative CT lesion seen on Fig. 3A, C.

Microbiological analysis on the resected tissue revealed acid-fast bacilli on microscopy, and positive Xpert MTB/RIF and culture results. Mtb grew from the caseous center, inner and outer walls of the cavity and a resected foci located ~ 3 cm from the cavity. DST revealed sensitivity to isoniazid, rifampin, and ethambutol.

Pathological examination of the resected lesion showed findings consistent with fibrocavernous tuberculosis. No postoperative complications were experienced, and the patient reinitiated first-line therapy via DOT on the 2nd postoperative day and was discharged on postoperative day 11.

**Fig. 5:**
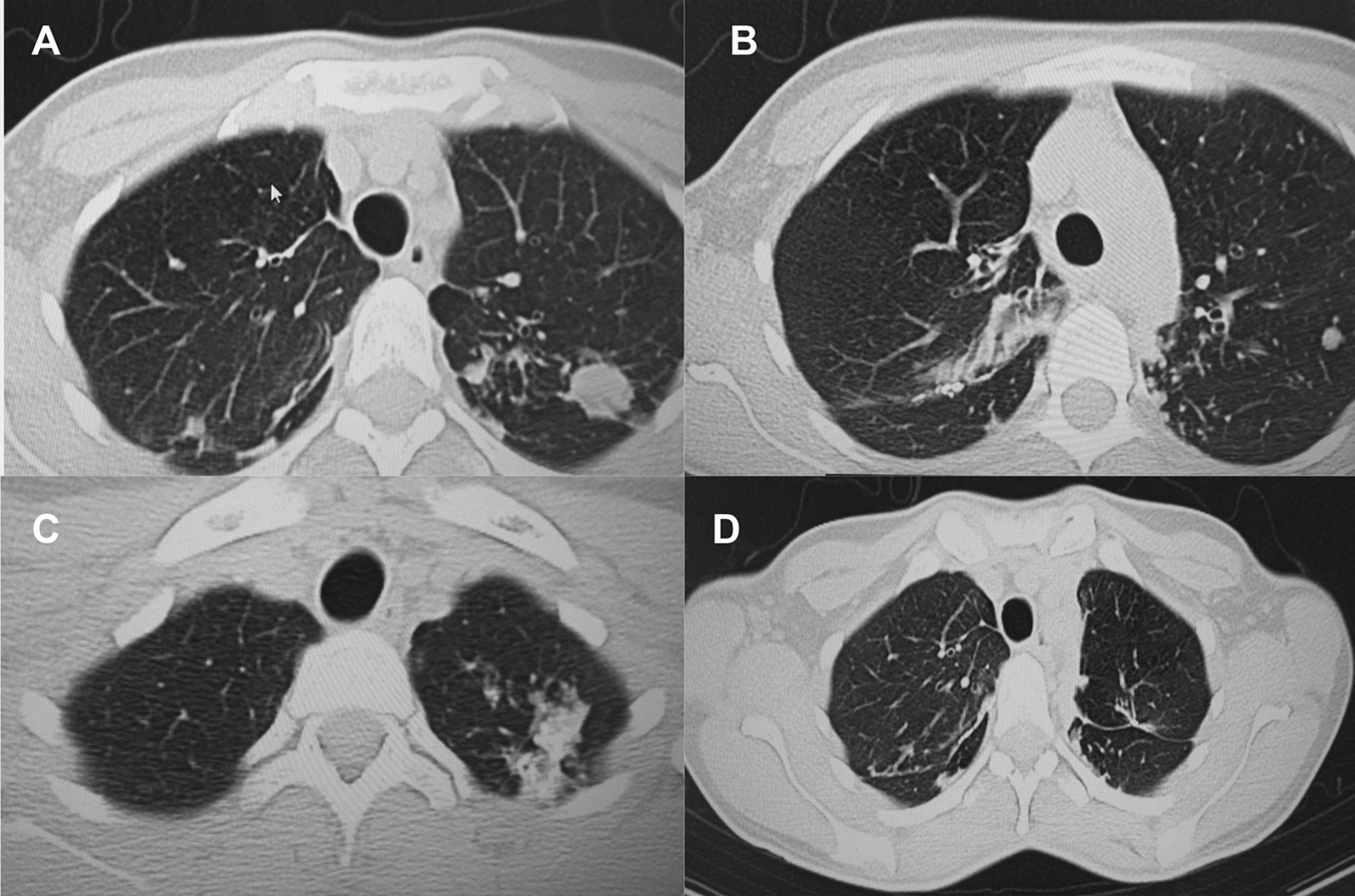
**A**–**C** Follow-up CT scan after first adjunctive surgery showing postoperative changes of the right lung and radiological changes in the left lung, that were unchanged compared to the initial CT. **D** Final CT scan showing normal postoperative changes with no cavities as previously seen

A follow up CT scan performed after 3 months showed postoperative changes in the right upper lobe, and an unchanged left lung (Fig. 5A–C). Based on the persistent conglomerate of tuberculomas and multiple small tuberculous foci, growth of Mtb from the previous surgical specimen, and the patient’s social situation (mother of three young children) a second surgery to optimize the chance of cure was recommended. The patient reported no symptoms, complaints, or functional disability before the surgery. Preoperative sputum testing found negative AFB smear microscopy and culture. The patient underwent the second operation on May 18, 2021, in which the S1, S2 and part of the S6 segment of the left lung were resected. Intraoperatively, moderate adhesions seen along with a dense palpable ~ 3 cm mass in the S1 and S2 region and a dense focus in S6.

Microbiological examinations performed on resected tissue revealed positive AFB smear microscopy and Xpert MTB/RIF results and a negative AFB culture. The pathological examination of the surgical samples indicated a variety of destructive changes in addition to ongoing inflammation. The gross specimen of S1 and S2 segments of the left lung showed fibrocavernous tuberculosis shown in Fig. 4B, which corresponds to the left lung lesion seen on the first preoperative CT in Figs. 3B and 5A in the second preoperative CT; the gross specimen of the S6 segment showed progressive tuberculoma seen in Fig. 4C, which corresponds to the left lung lesion seen on the first preoperative CT in Figs. 3D and 5C in the second preoperative CT.

There were no postoperative complications, and tuberculosis (TB) treatment was reinitiated. The patient successfully completed treatment with normalization of clinical and laboratory parameters and a clinical outcome of cure in September 2021, ~ 14 months after beginning treatment. The patient had reported near complete resolution of her symptoms, having a much better ability to perform her daily activities. The patient appreciated the effects surgery had on her recovery and was happy to have gone through that treatment route. A post treatment CT scan demonstrated postoperative changes in the upper segments of both lungs (Fig. 5D). Results from post treatment lung function testing were all within normal range.

## Discussion and conclusions

We present this case to highlight the heterogeneous nature of pulmonary tuberculosis and need for an individualized treatment approach, especially for patients with cavitary disease. Over the last decade, novel diagnostics, drugs, and treatment regimens have revolutionized TB management including a recent landmark clinical trial demonstrating an effective 4-month regimen for drug-susceptible TB [1]. The move towards shorter regimens is critical to improve treatment completion rates and help meet TB elimination goals. However, during a transition to shorter treatment durations it is imperative that clinicians remain aware of complex and severe pulmonary TB cases that may require longer durations of treatment and adjunctive therapies such as surgery. Supporting evidence comes from a recent landmark study finding persistent inflammation on imaging associated with finding Mtb mRNA in sputum after successful treatment and a meta-analysis demonstrating a hard-to-treat TB phenotype not cured with the standard 6 months of treatment [2, 5]. However, regarding recommendations for prolonging treatment beyond 6 months for drug-susceptible pulmonary tuberculosis, ATS/CDC/IDSA recommends (expert opinion) extended treatment for persons with cavitary disease and a positive 2 month culture (our patient would not have met this criteria); World Health Organization (WHO) does not recommend extended treatment for any persons with drug-susceptible TB [6, 7]. Accumulating evidence demonstrates surgical resection may be an effective adjunctive treatment in cases with cavitary disease [8–12]. Ultimately, a precision medicine approach towards TB will be able to identify patients who would benefit from short course therapy and those who would benefit from longer therapy and adjunctive treatment including surgery [13].

Mtb has a unique ability and propensity to induce cavities in humans with various studies showing cavitary lesions in ~ 30 to 85% of patients with pulmonary tuberculosis [14]. Lung cavities are more common in certain groups including patients with diabetes mellitus and undernutrition such as our patient who had a baseline BMI of 16.3 kg/m^2^ [15, 16]. Their presence indicates more advanced and severe pulmonary disease as evidenced by their association with worse clinical outcomes. Cavitary disease has been associated with higher rates of treatment failure, disease relapse, acquired drug resistance, and long term-term pulmonary morbidity [2, 17–19]. The impact of cavitary disease may be more pronounced in drug-resistant disease as shown in an observational study from our group which found a five times higher rate of acquired drug resistance and eight times higher rate of treatment failure among patients multidrug- or extensively drug-resistant cavitary disease compared to those without [20].

Mtb cavities are characterized by a fibrotic surface with variable vascularization, a lymphocytic cuff at the periphery followed by a cellular layer consisting of primarily macrophages and a necrotic center with foamy apoptotic macrophages and high concentrations of bacteria. Historically, each portion of the TB cavity has been conceptualized as concentric layers of a spherical structure due to its appearance on histologic cross-sections. However, recent studies using more detailed imaging techniques have shown most TB cavities exhibit complex structures with diverse, branching morphologies [21]. A dysregulated host immune response to Mtb is thought to contribute to the development of lung cavities, which may explain why cavitary lesions are seen less frequently among immunosuppressed patients including people living with Human Immunodeficiency Virus (HIV) [14]. The center of the TB cavity (caseum) is characterized by accumulation of pro-inflammatory lipid signaling molecules (eicosanoids) and reactive oxygen species, which result in ongoing tissue destruction, but do little to control Mtb replication [22]. Conversely, the cellular rim and lymphocytic cuff are characterized by a lower abundance of pro-inflammatory lipids and increases in immunosuppressive signals including elevated expression of TGF-beta and indoleamine-2,3-dioxygenase-1 [22]. The anti-inflammatory milieu within these TB cavity microenvironments impairs effector T cell responses, further limiting control of bacterial replication [23–25].

The combination of impaired cell-mediated immune responses with accumulation of inflammatory mediators at the rim of the caseum leads to ongoing tissue destruction with the potential for long-term pulmonary sequelae. Many with cavitary tuberculosis suffer chronic obstructive pulmonary disease after successful treatment and the risk may be greater in those with multidrug-resistant disease [3, 4]. This has led to research into adjunctive treatment with immune modulator therapies with a goal of mitigating the over-exuberant inflammatory response at the interior edge of the cavity to limit tissue damage. In a recent randomized clinical trial, patients with radiographically severe pulmonary tuberculosis treated with adjunctive everolimus or CC-11050 (phosphodiesterase inhibitor with anti-inflammatory properties) achieved better long-term pulmonary outcomes versus those who received placebo [26]. Such results suggest the inflammatory response can be modified with appropriate host-directed therapies to improve pulmonary outcomes, particularly in those with cavitary tuberculosis.

Tuberculosis cavities not only hinder an effective immune response, but also prevent anti-tuberculosis drugs from achieving sterilizing concentrations throughout the lesion and especially in necrotic regions. The necrotic center of cavitary lesions is associated with extremely high rates of bacilli (up to 10^9^ per milliliter), many of which enter a dormant state with reduced metabolic activity. Bacilli in this dormant state may be less responsive to the host immune response and exhibit phenotypic resistance to some anti-tuberculosis drugs thereby preventing sterilization and increasing chances of relapse [14, 27, 28]. The fact that the specimens from our patient’s second surgery were Xpert and AFB positive, but culture negative may indicate the presence of either dead bacilli or metabolically altered(dormant) bacilli that may be alive, but not culturable by standard techniques. Further, genomic sequencing studies have also found distinct strains of Mtb within different areas of the cavity that have varying drug-susceptibilities demonstrating cavities as a potential incubator for drug resistance [27, 29].

Emerging literature has started to elucidate the varying abilities of drugs to penetrate into cavitary lesions and the importance of adequate target site concentrations. One notable study found that decreasing tissue concentrations within resected cavitary TB lesions were associated with increasing drug phenotypic MIC values [30]. Innovative studies using MALDI mass spectrometry imaging have further demonstrated varied spatiotemporal penetration of anti-TB drugs in human TB cavities [31]. This study found rifampin accumulated within caseum, moxifloxacin preferentially at the cellular rim, and pyrazinamide throughout the lesion, demonstrating the need to consider drug penetration when designing drug regimens in patients with cavitary TB. Computational modeling studies have further demonstrated the importance of complete lesion drug coverage to ensure relapse-free cure [32]. Furthermore, clinical trials are now incorporating these principles into study design by (1) using radiological characteristics to determine treatment length and (2) incorporating tissue penetration into drug selection and regimen design [33, 34]. Beyond tissue penetration, varying drug levels and rapid INH acetylation status can also lead to suboptimal pharmacokinetics and poor clinical outcomes [35, 36]. As highlighted in a recent expert document, clinical standards to optimize and individualize dosing need to be developed to improve outcomes [37].

Available literature points to a benefit of adjunctive surgical resection particularly among patients with drug resistant tuberculosis. A meta-analysis of 24 comparative studies found surgical intervention was associated with favorable treatment outcomes among patients with drug-resistant TB (odds ratio 2.24, 95% CI 1.68–2.97) [38]. Additionally, an individual patient data meta-analysis found that partial lung resection (adjusted OR 3.9, 95% CI 1.5–5.9) but not pneumectomy was associated with treatment success [39]. In two observational studies, we have also found that adjunctive surgical resection was associated with high and improved outcomes compared to patients with cavitary disease not undergoing surgery and was associated with less reentry into TB care. It should be noted that all studies of surgical resection for pulmonary TB were observational studies, which may be subject to selection bias, and no clinical trials (very difficult to implement in practice) were conducted to provide more conclusive evidence. Based on available evidence, the WHO has provided guidance to consider surgery among certain hard to treat cases of both drug-susceptible and resistant cavitary disease [40]. Criteria for surgical intervention included (1) failure of medical therapy (persistent sputum culture positive for *M. tuberculosis*), (2) a high likelihood of treatment failure or disease relapse, (3) complications from the disease, (4) localized cavitary lesion, and (5) sufficient pulmonary function to tolerate surgery. For our patient, the severity of disease, lack of improvement of radiological imaging despite appropriate treatment, and high risk of relapse were the main indicators for surgery. Contraindications for surgery included a forced expiratory volume (FEV1) < 1000 mL, severe malnutrition, or patients at high risk for perioperative cardiovascular complications. With strict adherence to indications and contraindications for surgery, an acceptable level of postoperative complications are noted (5–17%) [4, 38]. Our results also demonstrate the safety of adjunctive surgery, as our post-operative complication rate (8%) was low with the majority being minor complications [41].

As our case highlights, patients with persistent cavitary disease at the end of treatment require close clinical follow up and a tailored, individualized plan to determine the best approach for disease elimination and cure. In certain cases, including those with persistent cavitary disease and end of treatment, and where available, surgical resection is an effective adjunctive treatment option that can reduce disease burden and aid anti-tuberculosis agents in providing a sterilizing cure. As we enter an era of welcomed new shorter treatment options for tuberculosis it is imperative for clinicians to be able to identify and recognize complicated TB cases that require prolonged treatment and potentially adjunctive surgery.

## Data Availability

Data sharing is not applicable to this article as no datasets were generated or analyzed during the current study.

## Abbreviations

AFB: Acid fast bacilli
ATS: American Thoracic Society
BMI: Body mass index
CDC: Center for Disease Control
CT: Computed tomography
DOT: Directly observed therapy
DST: Drug sensitive tuberculosis
ESR: Erythrocyte sedimentation rate
HIV: Human Immunodeficiency Virus
IDSA: Infectious Diseases Society of America
Mtb: *Mycobacterium tuberculosis*
TB: Tuberculosis
WHO: World Health Organization
